# The effects of BAFF on T lymphocytes in chronic obstructive pulmonary disease

**DOI:** 10.1186/s12931-020-01333-z

**Published:** 2020-03-11

**Authors:** Shupei Gao, Jinqing Chen, Jungang Xie, Jianmiao Wang

**Affiliations:** Department of Respiratory and Critical Care Medicine, Tongji Hospital, Tongji Medical College, Huazhong University of Science and Technology, 1095 Jiefang Road, Wuhan, 430030 China

**Keywords:** B cell activating factor belonging to the tumor necrosis factor family (BAFF), Chronic obstructive pulmonary disease, Inflammation, T lymphocytes

## Abstract

**Background:**

It has been reported that B cell activating factor belonging to the tumor necrosis factor family (BAFF) expression is increased in chronic obstructive pulmonary disease (COPD). However its role in this chronic inflammatory disease is not fully understood. Previous studies have suggested that BAFF also affects T cell function. We therefore investigated the effects of BAFF on T lymphocytes in COPD.

**Methods:**

BAFF was detected in the cells of sputum and the plasma. Peripheral blood mononuclear cells (PBMCs) were isolated from COPD patients and treated with BAFF or BAFF plus BR3-Fc (BAFF antagonist). The apoptosis of CD4^+^ cells and CD8^+^ cells was analyzed by flow cytometry. CD4^+^ cells and CD8^+^ cells were isolated from peripheral blood of COPD patients respectively and treated with BAFF or BAFF plus BR3-Fc. Interferon-γ (IFN-γ) and interleukin-4 (IL-4) were detected in the CD4^+^ cells, and perforin and granzyme B were detected in the CD8^+^ cells.

**Results:**

BAFF expression was increased in the cells of sputum and the plasma from COPD patients compared with control subjects. The plasma BAFF levels were inversely correlated with FEV_1_ percentage of predicted in patients with COPD. BAFF did not significantly alter the apoptosis of CD4^+^ cells, however it significantly inhibited the apoptosis of CD8^+^ cells from COPD patients. BAFF increased IFN-γ expression in the CD4^+^ cells from COPD patients, while it did not significantly alter the expresson of IL-4 in these cells. BAFF increased the expression of perforin and granzyme B in the CD8^+^ cells from COPD patients.

**Conclusions:**

Our findings indicate that BAFF may be involved in the inflammatory response in COPD via affecting T lymphocytes, suggesting a possible role of BAFF in the pathogenesis of COPD.

## Background

Chronic obstructive pulmonary disease (COPD) is a common disease characterized by persistent respiratory symptoms and airflow limitation, which is a leading cause of morbidity and mortality worldwide [1]. The prevalence and burden of COPD are projected to increase over the coming decades due to continued exposure to risk factors and aging of the world’s population [2]. The lung inflammation caused by cigarette smoke or other noxious particles induces tissue destruction and airway fibrosis, leading to the gas trapping and progressive airflow limitation [3].

The pathological changes observed in COPD include chronic inflammation and structural changes with increased numbers of specific inflammatory cell types in different parts of the lung [4]. Previous studies have shown that the number of pulmonary CD8^+^ T cells in COPD increases substantially with higher stages of airflow limitation, and reduced apoptosis of CD8^+^ cells may contribute to the accumulation of these cells in the lung [5]. CD8^+^ T cells release proteolytic enzymes such as perforin and granzymes, which cause cell death of structural cells by apoptosis or necrosis [6]. Numbers of CD4^+^ T helper 1 (Th1) cells are also raised in the airways and lungs of COPD, which produce interferon-γ (IFN-γ) and promote accumulation of inflammatory cells to the lung. Lung lymphocytes in patients with COPD have higher percentages of CD4^+^ Th1 cells and secrete more IFN-γ than in control smokers [7]. These findings suggest that T lymphocytes including CD8^+^ and CD4^+^ T cells play an important role in the pathogenesis of COPD.

B cell activating factor belonging to the tumor necrosis factor family (BAFF) is an important cytokine for B cell survival and maturation [8], which is expressed by many cell types including macrophages, dendritic cells and epithelial cells [9]. It binds to three receptors on B cells including BAFF receptor (BR3), transmembrane activator and calcium modulator cyclophilin ligand interactor (TACI) and B cell maturation antigen (BCMA). Overexpression of BAFF is associated with autoimmune diseases in humans and mice [10, 11]. It has been reported that BAFF expression is increased in the lung of patients with COPD, mainly in alveolar macrophages and lymphoid follicles, and BAFF-positive macrophages are inversely related to FEV_1_ in COPD [12]. Later studies have shown that recombinant BAFF blocks cigarette smoke extract induced B cell apoptosis and antagonizing BAFF in cigarette smoke exposed mice attenuates pulmonary inflammation and alveolar destruction [13, 14]. These findings suggest that BAFF might be associated with increasing lung function decline in COPD.

Previous studies have suggested that BAFF also affects T cell function through binding to the receptor BR3 and augments certain Th1 associated inflammatory responses [15, 16]. However, it is not clear whether BAFF affects T lymphocytes in COPD. In the present study, we utilized in vitro experiments to investigate the effects of BAFF on CD8^+^ and CD4^+^ cells from the peripheral blood of COPD patients.

## Methods

### Subjects

COPD patients aged 40 to 80 years old were recruited from the out-patient department of Tongji Hospital, Tongji Medical College, Huazhong University of Science and Technology, Wuhan, China, between 2018 and 2019. All patients were diagnosed with COPD based on clinical history, physical examination, chest radiograph and spirometry according to the Global Initiative for Chronic Obstructive Lung Disease (GOLD) criteria [1]. Inclusion critiria are as follows: males aged between 40 to 80 years old with a history of at least 20 pack-years of smoking. Exclusion criteria are as follows: chronic respiratory diseases such as asthma, active tuberculosis, bronchiectasis and interstitial lung disease; cardiac, hepatic or renal failure; malignant diseases; autoimmune diseases; and current oral steroid therapy. All COPD patients were examined in stable condition. Nonsmokers without COPD and smokers without COPD were recruited from the health screening center of our hospital as control subjects. The exclusion criteria mentioned above were also used to screen the control subjects. The study was approved by the hospital ethics committees, and all subjects gave written informed consent.

### Pulmonary function tests

Forced vital capacity and forced expiratory volume in the first second (FEV_1_) were obtained from the flow-volume curve using an appropriately calibrated spirometer (Jaeger, Wurzburg, Germany) before and 20 min after salbutamol inhalation. Three technically acceptable measurements were performed on each patient, and the highest value was selected and expressed as a percentage of reference values. The predicted FEV_1_ was calculated using the following prediction equations recommended by the American Thoracic Society/European Respiratory Society Task Force 2005 [17] (Predicted FEV_1_ = 4.30 × height in meters-0.029 × age-2.49).

### Sample collection

Sputum and heparinized peripheral venous blood samples were collected from nonsmokers, smokers and COPD patients. Sputum induction with hypertonic saline was performed as previously described [18]. Sputum plugs were separated from sputum, and dithiothreitol was used to disperse mucus. Cytospin preparations for immunocytochemistry were made with centrifugation at 980 rpm for 8 mintues. Plasma was separated from fresh heparinized blood, and the blood cells were used for peripheral blood mononuclear cell (PBMC), CD4^+^ cells or CD8^+^ cells isolation.

### Immunocytochemistry

Slides were washed with phosphate buffered saline (PBS) and incubated with 3% H_2_O_2_ for 10 min. After incubation with 5% bovine serum albumin (BSA) for 45 min, BAFF was localized using the rabbit polyclonal anti-BAFF antibody (Abcam, Cambridge, MA, USA). The bound antibodies were detected with horseradish peroxidase (HRP)-conjugated secondary antibody (Gene Tech, Shanghai, China), then 3-amino-9-ethylcarbazole (Boster, Wuhan, China) was added for the detection and haematoxylin (Boster, Wuhan, China) was used for the nucleus counterstain.

### Enzyme-linked immunosorbent assay

The BAFF protein levels in plasma were determinated using enzyme-linked immunosorbent assay (ELISA) kits (R&D Systems, Minneapolis, MN, USA) according to the manufacturer’s instructions. The protein levels of IFN-γ and IL-4 in the supernatant of CD4^+^ cells culture were detected using ELISA kits (R&D Systems, Minneapolis, MN, USA) according to the manufacturer’s instructions.

### Cells isolation

PBMCs were isolated from heparinized peripheral venous blood of COPD patients using density centrifugation. Briefly, the fresh peripheral blood was centrifugated at 1500 rpm for 10 min, then the supernatant containing leukocytes was mixed with PBS, and was carefully layered onto Ficoll-Hypaque gradient (Dakewe Biotech, Shenzhen, China). PBMCs layer was obtained after centrifugation, cells were washed and resuspended for cell culture or for CD4^+^ or CD8^+^ cells isolation. CD4^+^ and CD8^+^ cells of COPD patients were separated from the PBMCs using MagCellect Human CD4^+^/CD8^+^ T Cell Isolation Kits (R&D Systems, Minneapolis, MN, USA) according to the manufacturer’s instructions.

### Cell culture

The isolated PBMCs were washed with pre-cold PBS and cultured with supplemented RPMI 1640 medium in 24-well plates. Cells were stimulated with rhBAFF (20 ng/ml, R&D Systems, Minneapolis, MN, USA) or a combination of rhBAFF and BR3-Fc (100μg/ml, R&D Systems, Minneapolis, MN, USA) at 37 °C with 5% CO_2_ for 48 h. Cells were harvested for assessment of apoptosis of CD4^+^ and CD8^+^ cells using flow cytometry. Isolated CD4^+^ or CD8^+^ cells were cultured with supplemented RPMI 1640 medium and phytohemagglutinin (Sigma-Aldrich and Merck KGaA, Darmstadt, Germany) in 24-well plates. Cells were stimulated with rhBAFF or a combination of rhBAFF and BR3-Fc at 37 °C with 5% CO_2_ for 72 h. For CD4^+^ cells culture, the supernatant and cells were seperately obtained for the detection of IL-4 and IFN-γ expression. For CD8^+^ cells culture, the cells were harvested for the detection of mRNA expression of perforin and granzyme B.

### Flow cytometry

PBMCs were harvested for detection of apoptosis of CD4^+^ and CD8^+^ cells using flow cytometry. Briefly, after blocking, cells were incubated on ice with FITC-conjugated anti-CD4 antibody (Biolegend, San Diego, CA, USA) and PE-conjugated anti-CD8 antibody (Biolegend, San Diego, CA, USA) for 30 min in the dark. The cells were washed and incubated with APC-conjugated annexin V (Biolegend, San Diego, CA, USA) for 15 min in the dark and were analyzed.

### Quantitative polymerase chain reaction

Total RNA was isolated from the CD4^+^ cells and CD8^+^ cells and was reverse-transcribed into cDNA using PrimeScript RT Reagent Kit (Takara, Shiga, Japan). Quantitative polymerase chain reaction (PCR) was performed using a Bio-Rad CFX Connect Real-Time System (Bio-Rad, Hercules, CA, USA) with SYBR Premix Ex Taq (Takara, Shiga, Japan) and the specific primers. The primer sequences were as follows: β-actin (forward, GCAAGCAGGACTATGACGAG and reverse, CAAATAAAGCCATGCCAATC),

IFN-γ (forward, TTCTTACAACACAAAATCAAATCA and reverse, TCAACAAAGCTGATACTCCA), IL-4 (forward, ATGGGTCTCACCTCCCAACT and reverse, GATGTCTGTTACGGTCAACTCG), perforin (forward, CCCAGTGGACACACAAAGGTT and reverse, TCGTTGCGGATGCTACGAG), granzyme B (forward, CCCTGGGAAAACACTCACACA and reverse, CACAACTCAATGGTACTGTCGT). The relative mRNA expression was determined using the 2^-ΔΔCt^ methods with β-actin as endogenous control.

### Statistical analysis

Results were expressed as mean ± SEM unless otherwise specified. D’Agostino and Pearson omnibus normality test was used to determine whether the data were normally distributed. Data that were normally distributed were assessed for significance by Student’s t-test or ANOVA as appropriate. Data that were not normally distributed were assessed for significance using the Mann-Whitney U-test or the Kruskal-Wallis test with Dunn’s posttest for multiple comparisons as appropriate. The correlations were analyzed by Pearson’s correlation. Statistical analysis was performed using Prism version 6 (GraphPad). A two-sided *p*-value < 0.05 was considered to be statistically significant.

## Results

### Characteristics of subjects

The characteristics of subjects are shown in Table 1. Totally, 94 subjects were recruited to the study including 16 nonsmokers without COPD, 36 smokers without COPD and 42 COPD patients. All of them were male, and there were no significant differences in age and body mass index among the three groups. There was no significant difference in smoking index between the smokers and the patients with COPD. FEV_1_%predicted (FEV_1_%pred), FEV_1_/forced vital capacity (FVC) and FVC%pred were significantly lower in patients with COPD than those in the smokers and nonsmokers.

**Table 1:** Clinical characteristics of subjects in this study

	Nonsmoker	Smoker	COPD
Subjects	16	36	42
Age yrs	58.6 ± 2.2	60.2 ± 1.1	63.7 ± 1.2
Male	16(100%)	36(100%)	42(100%)
Smoking index p.y	0	47.8 ± 4.3	48.6 ± 4.0
BMI kg/m^2^	23.1 ± 0.5	23.0 ± 0.3	22.1 ± 0.3
FVC % pred	108.3 ± 3.5	114.2 ± 2.5	90.6 ± 2.8^**^
FEV_1_/FVC%	81.0 ± 1.3	77.3 ± 1.0	52.0 ± 2.0^**^
FEV_1_% pred	109.7 ± 3.2	109.2 ± 2.6	61.1 ± 3.5^**^

Values are numbers (%) or mean ± SEM; *COPD* Chronic Obstructive Pulmonary Disease, *p.y* pack-yrs, *BMI* body mass index, *FVC* forced vital capacity, *FEV*_*1*_ forced expiratory volume in 1 sec, *% pred* % predicted; **: *P* < 0.01 versus nonsmoker and smoker

### BAFF expression in the cells of sputum

**Fig. 1:**
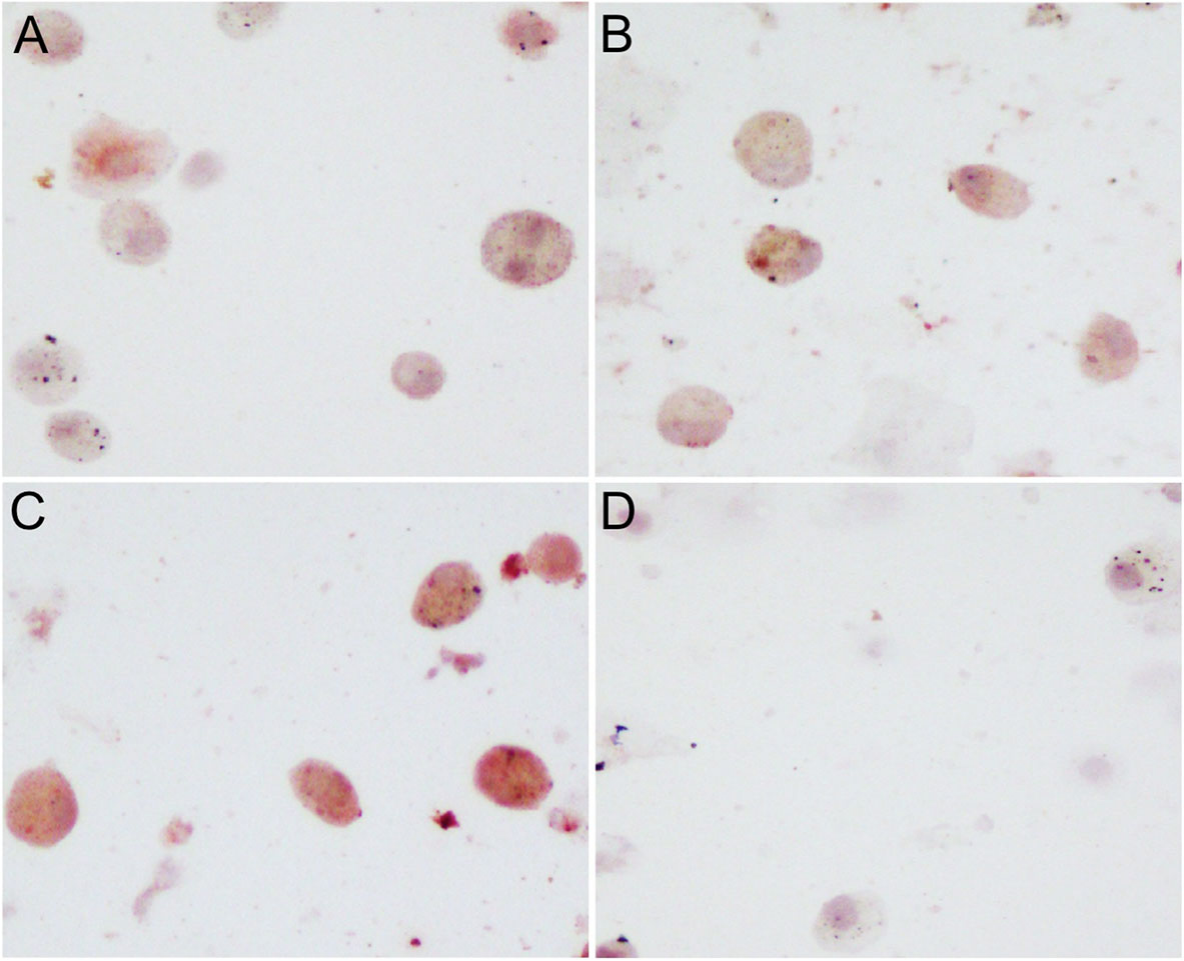
B cell activating factor (BAFF) expression in the cells of sputum from the subjects. The representative immunocytochemistry images of BAFF staining in the cells of sputum from a nonsmoker without COPD (**a**), a smoker without COPD (**b**) and a COPD patient (**c**) are shown, and the negative control is also shown (**d**). Original magnification, ×200

To investigate BAFF expression in the cells of sputum from nonsmokers, smokers and COPD patients, sputum samples were obtained and BAFF expression was detected using immunocytochemistry. The representative results were shown in Fig. 1. The expression of BAFF in the cells (mainly macrophages) of sputum from COPD patients was increased compared with smokers and nonsmokers.

### BAFF expression in the plasma

**Fig. 2:**
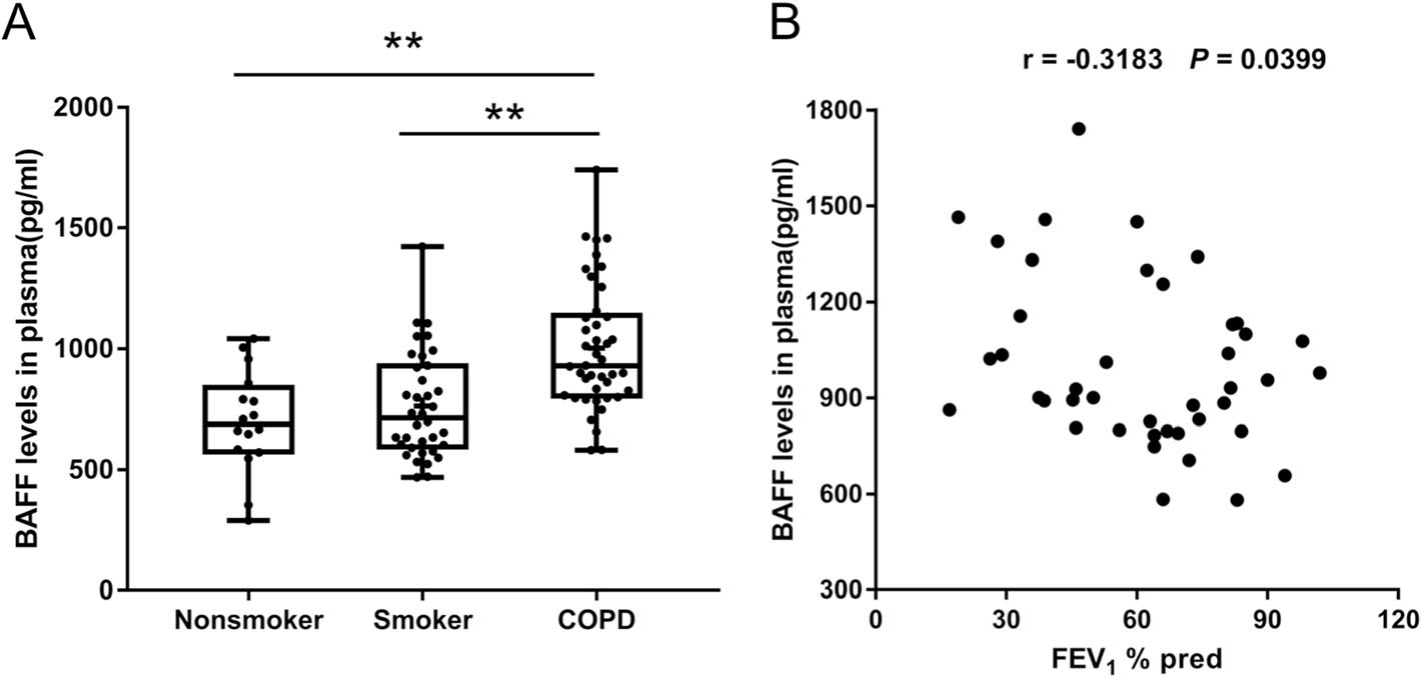
B cell activating factor (BAFF) expression in the plasma from the subjects. The expression levels of BAFF in the plasma from nonsmokers without COPD (*n* = 16), smokers without COPD (*n* = 36) and COPD patients (*n* = 42) are shown (**a**). The data are presented as median (P25 quartile, P75 quartile). The correlation between the plasma BAFF expression levels and forced expiratory volume in 1 sec (FEV_1_)% predicted in COPD patients (*n* = 42) is shown (**b**). **P < 0.01

To assess BAFF expression in the palsma of COPD patients, peripheral venous blood samples from nonsmokers, smokers and patients with COPD were obtained and ELISA was performed. BAFF expression in plasma was significantly increased in COPD patients compared with nonsmokers and smokers (Fig. 2a). We also analyzed the association between plasma BAFF expression and pulmonary function,

and found that plasma BAFF levels were significantly correlated with FEV_1_%pred in patients with COPD (Fig. 2b).

### Effects of BAFF on the apoptosis of CD4^+^ cells

**Fig. 3:**
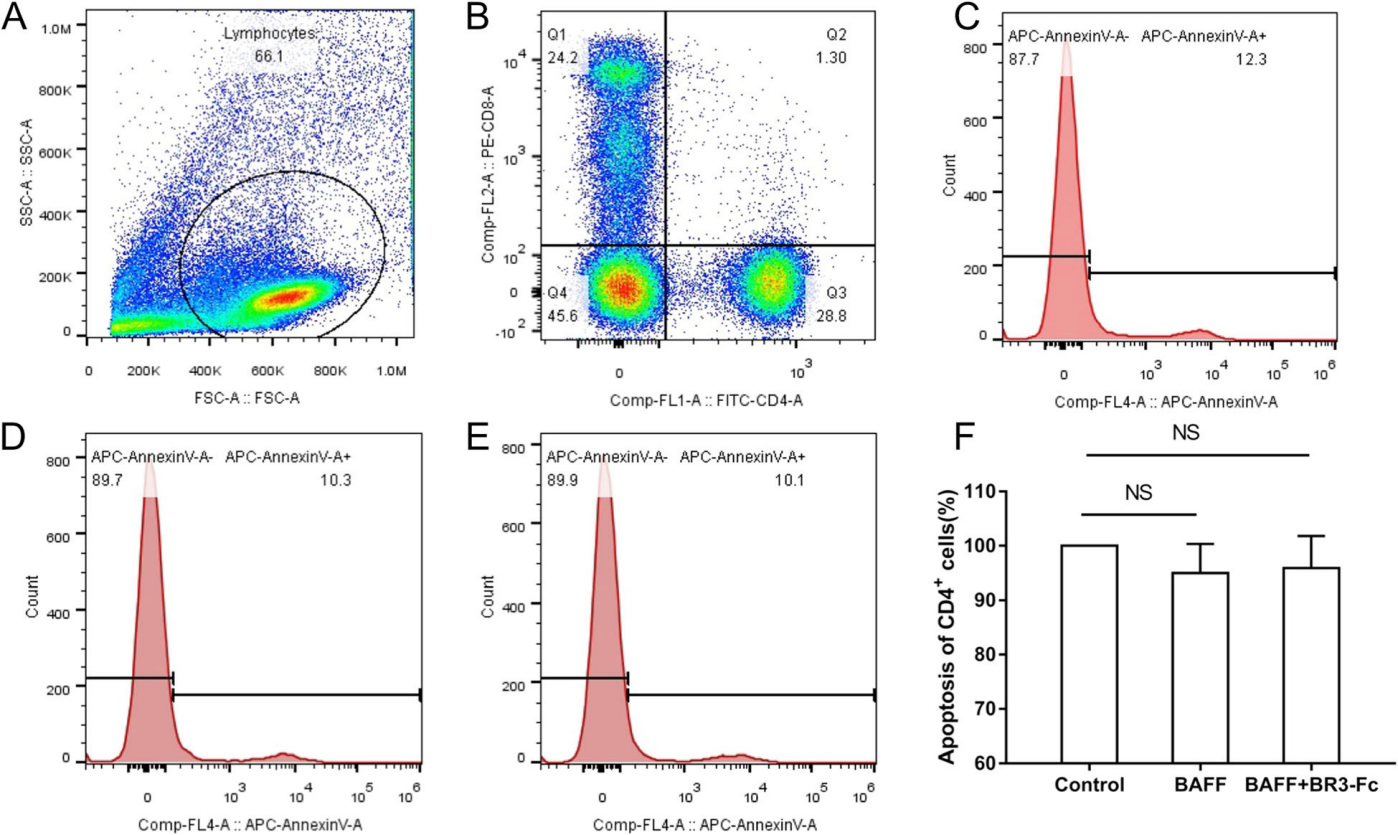
Effects of B cell activating factor (BAFF) on the apoptosis of CD4^+^ cells from COPD patients. Peripheral blood mononuclear cells (PBMCs) isolated from COPD patients were treated with recombinant human BAFF (rhBAFF) or rhBAFF plus BR3-Fc (BAFF antagonist) and the apoptosis of CD4^+^ cells was analyzed by flow cytometry. The gating strategy for identifying CD4^+^ cells and CD8^+^ cells is shown (**a** and **b**). The representative data of apoptosis of CD4^+^ cells from a COPD patient that were treated with control (**c**), rhBAFF (**d**) or rhBAFF plus BR3-Fc (**e**) are shown. The apoptosis of CD4^+^ cells from COPD patients (*n* = 13) treated with control, rhBAFF or rhBAFF plus BR3-Fc is shown (**f**). Data are presented relative to the control which is set at 100%. NS, nonsignificant

To investigate the effects of BAFF on the apoptosis of CD4^+^ lymphocytes in COPD, PBMCs from 13 COPD patients were cultured and treated with rhBAFF or a combination of rhBAFF and BR3-Fc (BAFF antagonist). The characteristics of 13 COPD patients are shown in Table 2. The apoptosis of CD4^+^ cells was analyzed using flow cytometry. The gating strategy was shown in Fig. 3a and b. We found that BAFF did not significantly alter the apoptosis of CD4^+^ cells in COPD (Fig. 3c, d, e and f).

**Table 2:** Characteristics of patients for PBMCs isolation and stimulation

Patient number	Sex	Age yrs.	BMI kg/m^2^	Smoking index p.y	FEV_1_% pred
1	male	64	25.0	50	46.7
2	male	68	18.5	150	33.2
3	male	72	21.3	30	46.0
4	male	59	22.6	20	63.0
5	male	54	24.5	20	50.0
6	male	55	26.7	37	67.0
7	male	72	22.9	30	40.6
8	male	64	24.2	80	22.0
9	male	74	23.5	50	74.3
10	male	69	23.7	40	33.9
11	male	72	22.2	60	64.2
12	male	71	21.1	45	26.3
13	male	57	25.6	20	36.9

*PBMCs* peripheral blood mononuclear cells, *BMI* body mass index ,*p.y* pack-yrs, *FEV*_*1*_ forced expiratory volume in 1 sec, *%pred* %predicted

### Effects of BAFF on the apoptosis of CD8^+^ cells

**Fig. 4:**
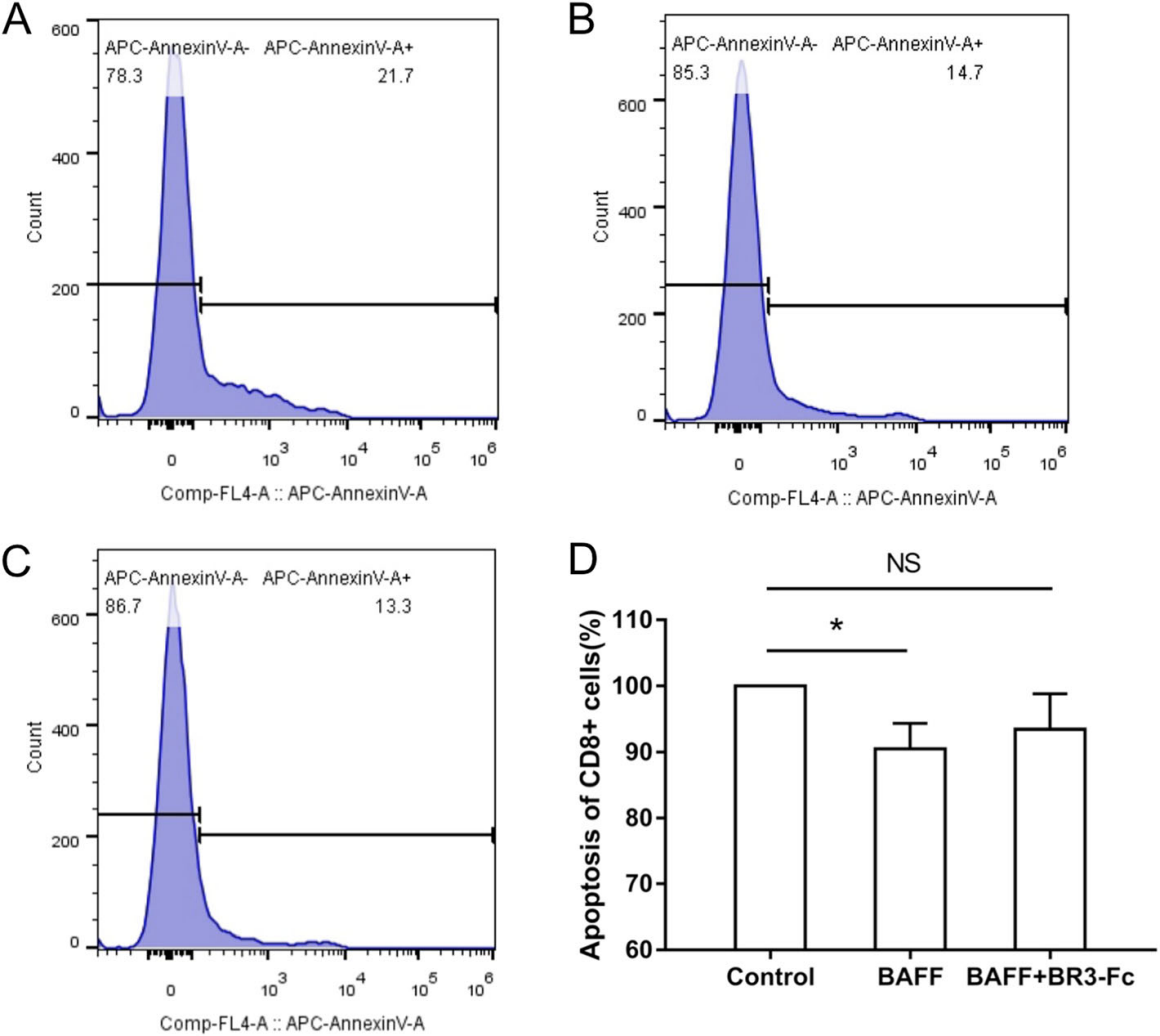
Effects of B cell activating factor (BAFF) on the apoptosis of CD8^+^ cells from COPD patients. Peripheral blood mononuclear cells (PBMCs) isolated from COPD patients were treated with recombinant human BAFF (rhBAFF) or rhBAFF plus BR3-Fc (BAFF antagonist) and the apoptosis of CD8^+^ cells was analyzed by flow cytometry. The representative data of apoptosis of CD8^+^ cells from a COPD patient that were treated with control (**a**), rhBAFF (**b**) or rhBAFF plus BR3-Fc (**c**) are shown. The apoptosis of CD8^+^ cells from COPD patients (*n* = 13) treated with control, rhBAFF or rhBAFF plus BR3-Fc is shown (**d**). Data are presented relative to the control which is set at 100%. *P < 0.05. NS, nonsignificant

To investigate the effects of BAFF on the apoptosis of CD8^+^ lymphocytes in COPD, PBMCs from 13 COPD patients were cultured and treated with rhBAFF or a combination of rhBAFF and BR3-Fc. The characteristics of these 13 COPD patients are shown in Table 2. The apoptosis of CD8^+^ cells was analyzed using flow cytometry. We found that BAFF significantly inhibited the apoptosis of CD8^+^ cells in COPD (Fig. 4).

### Effects of BAFF on cytokine expression of CD4^+^ cells

**Fig. 5:**
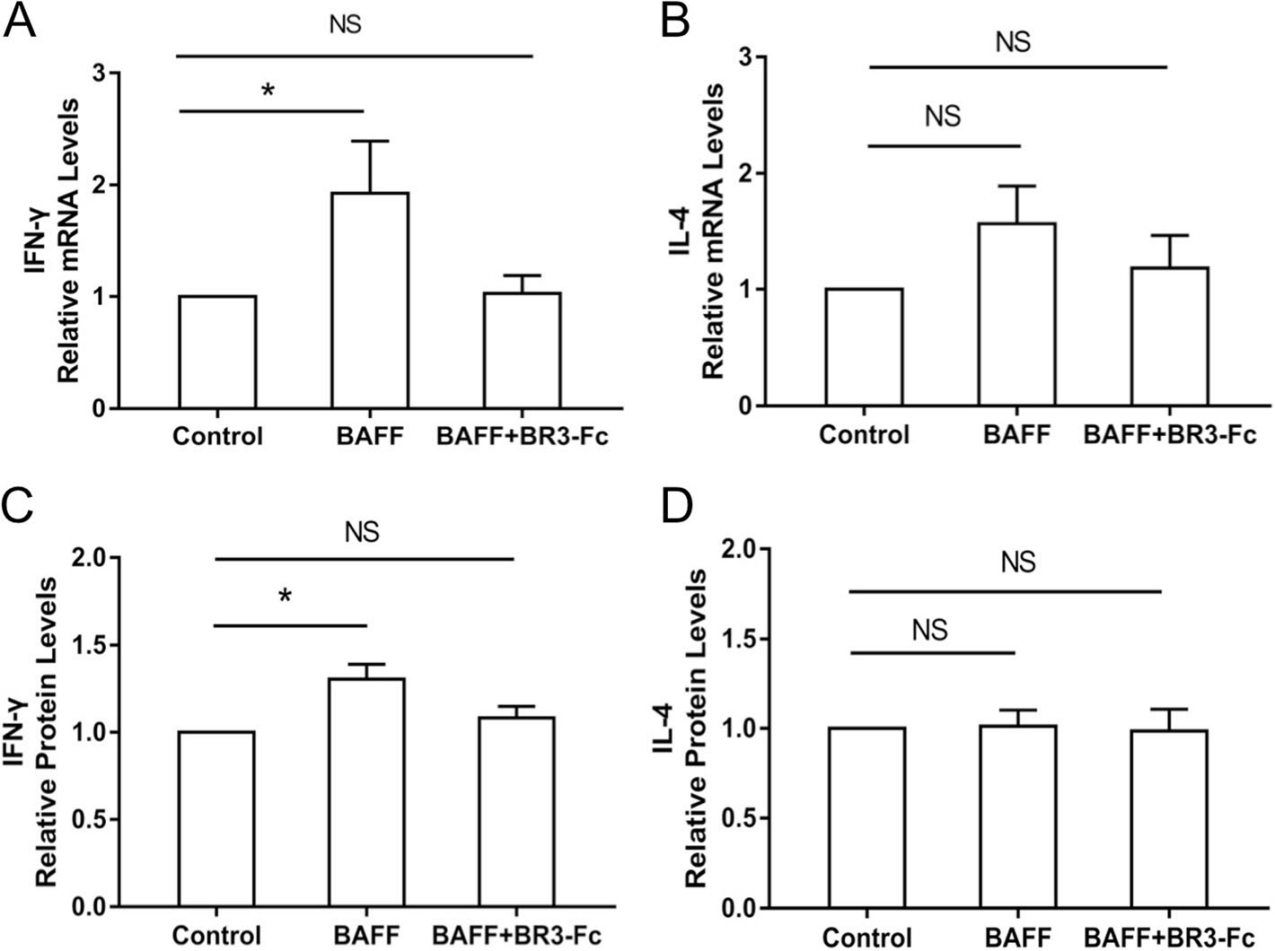
Effects of B cell activating factor (BAFF) on cytokine expression of CD4^+^ cells from COPD patients. CD4^+^ cells isolated from COPD patients were treated with control, recombinant human BAFF (rhBAFF) or rhBAFF plus BR3-Fc (BAFF antagonist) and the expression levels of interferon-γ (IFN-γ) and interleukin-4 (IL-4) were detected. The relative mRNA levels of IFN-γ (**a**) and IL-4 (**b**) in the CD4^+^ cells from COPD patients (*n* = 12) are shown. The relative protein levels of IFN-γ (**c**) and IL-4 (**d**) in the supernatant of CD4^+^ cells from COPD patients (*n* = 12) are shown. *P < 0.05. NS, nonsignificant

To study the effects of BAFF on the cytokine expression of CD4^+^ cells including IFN-γ and IL-4, CD4^+^ cells were isolated from the PBMCs of 12 COPD patients and cultured. The characteristics of these 12 COPD patients are shown in Table 3. CD4^+^ cells were treated with rhBAFF or a combination of rhBAFF and BR3-Fc. The mRNA levels of IFN-γ and IL-4 in the cells and their protein levels in the supernatant were detected. We found that the mRNA levels of IFN-γ in the CD4^+^ cells were increased significantly after BAFF treatment (Fig. 5a), while BAFF did not significantly alter the mRNA levels of IL-4 (Fig. 5b). The protein levels of IFN-γ in the supernatant were also increased significantly after BAFF treatment (Fig. 5c), while BAFF did not significantly alter the protein levels of IL-4 in the supernatant (Fig. 5d).

**Table 3:** Characteristics of patients for CD4^+^ cells isolation and stimulation

Patient number	Sex	Age yrs.	BMI kg/m^2^	Smoking index p.y	FEV_1_% pred
1	male	72	22.9	30	40.6
2	male	64	24.2	80	22.0
3	male	74	23.5	50	74.3
4	male	69	23.7	40	33.9
5	male	72	22.2	60	64.2
6	male	71	21.1	45	26.3
7	male	57	25.6	20	36.9
8	male	64	16.7	30	16.1
9	male	48	22.5	30	60.3
10	male	66	28.0	40	25.0
11	male	67	19.3	40	45.0
12	male	54	26.3	80	33.8

*BMI* body mass index, *p.y* pack-yrs, *FEV*_*1*_ forced expiratory volume in 1 sec, *%pred* %predicted

### Effects of BAFF on perforin and granzyme B expression of CD8^+^ cells

**Fig. 6:**
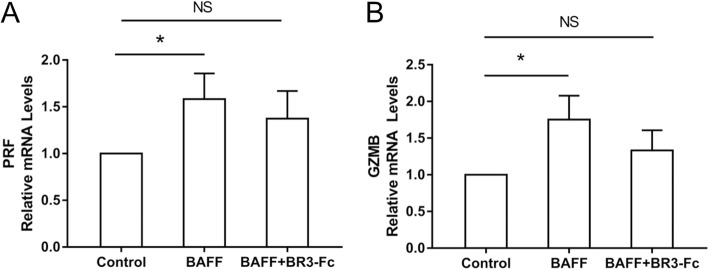
Effects of B cell activating factor (BAFF) on perforin and granzyme B expression of CD8^+^ cells from COPD patients. CD8^+^ cells isolated from COPD patients were treated with control, recombinant human BAFF (rhBAFF) or rhBAFF plus BR3-Fc (BAFF antagonist) and the expression levels of perforin (PRF) and granzyme B (GZMB) were detected. The relative mRNA levels of PRF (**a**) and GZMB (**b**) in the CD8^+^ cells from COPD patients (*n* = 10) are shown. *P < 0.05. NS, nonsignificant

To study the effects of BAFF on the perforin and granzyme B expression of CD8^+^ cells, CD8^+^ cells were isolated from the PBMCs of 10 COPD patients and cultured. The characteristics of these 10 COPD patients are shown in Table 4. CD8^+^ cells were treated with rhBAFF or a combination of rhBAFF and BR3-Fc. The mRNA levels of perforin and granzyme B in the cells were detected. We found that the mRNA levels of perforin and granzyme B in the CD8^+^ cells were both increased significantly after BAFF treatment (Fig. 6).

**Table 4:** Characteristics of patients for CD8^+^ cells isolation and stimulation

Patient number	Sex	Age yrs.	BMI kg/m^2^	Smoking index p.y	FEV_1_% pred
1	male	48	22.5	30	60.3
2	male	67	19.3	40	45.0
3	male	54	26.3	80	33.8
4	male	64	16.7	30	16.1
5	male	56	25.9	30	34.8
6	male	67	24.5	40	41.2
7	male	70	22.3	25	33.3
8	male	67	18.4	32	33.4
9	male	75	26.0	25	43.1
10	male	48	27.6	20	54.3

*BMI* body mass index, *p.y* pack-yrs, *FEV*_*1*_ forced expiratory volume in 1 sec, *%pred* %predicted

## Discussion

BAFF has been extensively studied in autoimmune diseases such as systemic lupus erythematosus and rheumatoid arthritis [19]. Several studies have suggested that BAFF may be implicated in the pathogenesis of COPD including our previous research using mouse models [20], however its role in this chronic inflammatory disease is not fully understood. Considering that BAFF also regulates T cell function, in the present study, we mainly investigated its effect on T lymphocytes in COPD.

Using immunocytochemistry, we show that BAFF expression in the cells (mainly macrophages) of sputum from COPD patients is increased compared with smokers and nonsmokers, which is consistent with the findings of previous clinical studies [12, 13]. We also show that BAFF expression in the plasma is significantly increased in COPD patients compared with nonsmokers and smokers. Further analysis suggests that plasma BAFF levels are inversely correlated with FEV_1_%pred in patients with COPD. These results imply that BAFF may play an important role in the inflammatory response in COPD and plasma BAFF may serve as a biomarker for disease severity.

The abnormal inflammatory response in the small airways and alveoli in COPD involves many inflammatory cells including neutrophils, macrophages and T lymphocytes [21]. T lymphocytes are increased in patients with COPD and the number of T lymphocytes/mm^3^ of lung is correlated with the extent of emphysema [22]. Different T cell subsets have been identified in the pathogenesis of this disease including CD8^+^ and CD4^+^ T cells, and the former predominates over the latter in the airways and lung parenchyma [23]. Previous studies have shown that there is a significant negative association between the CD8^+^ T cell subset and FEV_1_ percentage of predicted in the chronic bronchitic smokers [24]. Later studies also suggest that reduced apoptosis of CD8^+^ cells may contribute to the accumulation of these cells in the lung [5]. BAFF has been shown to promote the survival of CD8^+^ cells in immune thrombocytopenia patients [25], however the effect of BAFF on the apoptosis of T lymphocytes in COPD has not been studied yet. In the present study, we show that BAFF significantly inhibits the apoptosis of CD8^+^ cells in COPD, which might be one of the ways that BAFF participates in the pathogenesis of this disease. However we also show that BAFF does not significantly alter the apoptosis of CD4^+^ cells in COPD, suggesting that BAFF might have different effects on different T cell subsets.

Cytotoxic CD8^+^ T cells can induce apoptosis of target cells including bronchial epithelial cells through multiple mechanisms including the granzyme mediated pathway [26]. Granzyme B and perforin are stored in cytoplasmic secretory granules of these cytotoxic cells, and released into the intercellular space following adhesion to the target cell. Perforin forms transmembrane pores in the target cell, facilitating the entry of granzyme B and induction of apoptosis by activation of caspases [27]. It has been shown that the expression of granzyme B and perforin is increased in T cells from COPD patients [28, 29]. In the present study, we show that the expression levels of perforin and granzyme B in the CD8^+^ cells from COPD patients are both increased significantly after BAFF stimulation, suggesting that BAFF may affect the granzyme/perforin mediated pathway in COPD. This might be another way that BAFF participates in the pathogenesis of this disease.

CD4^+^ T cells that accumulate in the airways and lungs of COPD patients mainly produce IFN-γ and therefore have a Th1 phenotype [30]. Previous studies have suggested that intracellular Th1 proinflammatory cytokine production is increased in peripheral blood, bronchoalveolar lavage and intraepithelial T cells of COPD subjects, and the number of IFN-γ positive lymphocytes is inversely correlated with FEV_1_%pred [31, 32]. It has been shown that there is an increased percentage of IFN-γ producing cells and a decreased percentage of IL-4 producing cells among peripheral blood CD4^+^ T cells from the patients with COPD compared with control subjects [33]. In the present study, we show that the expression of IFN-γ in the CD4^+^ cells from COPD patients is increased significantly after BAFF treatment, however BAFF does not significantly alter the expression of IL-4 in these cells. The results suggest that BAFF may augment Th1 associated inflammatory responses in COPD.

In summary, we report that BAFF expression is increased in the cells of sputum and the plasma from COPD patients and the plasma BAFF levels are inversely correlated with FEV_1_%pred in COPD; BAFF inhibits the apoptosis of CD8^+^ cells and increases the expression of perforin and granzyme B in these cells from COPD patients; BAFF increases IFN-γ but not IL-4 expression in the CD4^+^ cells from COPD patients. Our findings indicate that BAFF may be involved in the inflammatory response in COPD via affecting T lymphocytes, suggesting a possible role of BAFF in the pathogenesis of COPD.

## Data Availability

Not applicable.

## Abbreviations

APC: Allophycocyanin
BAFF: B cell activating factor belonging to the tumor necrosis factor family
BCMA: B cell maturation antigen
BSA: Bovine serum albumin
COPD: Chronic obstructive pulmonary disease
ELISA: Enzyme-linked immunosorbent assay
FEV_1_: Forced expiratory volume in one second
FITC: Fluorescein isothiocyanate
FVC: Forced vital capacity
GOLD: Global Initiative for Chronic Obstructive Lung Disease
IFN-γ: Interferon-γ
IL-4: Interleukin 4
PBMC: Peripheral blood mononuclear cell
PBS: Phosphate buffered saline
PCR: Polymerase chain reaction
PE: Phycoerythrin
TACI: Transmembrane activator and calcium modulator cyclophilin ligand interactor
